# Determining Factors Influencing Collegiate Players’ Intention to Pursue a Professional Career

**DOI:** 10.3390/sports12040098

**Published:** 2024-03-30

**Authors:** Mathew Gerald D. C. Lebria, Cymond R. Ochoa, Jasmin Marie P. Tionloc, Ardvin Kester S. Ong, Josephine D. German

**Affiliations:** 1 School of Industrial Engineering and Engineering Management, Mapúa University, 658 Muralla St., Intramuros, Manila 1002, Philippines; mgdlebria@mymail.mapua.edu.ph (M.G.D.C.L.); crochoa@mymail.mapua.edu.ph (C.R.O.); jmptionloc@mymail.mapua.edu.ph (J.M.P.T.);; 2 E.T. Yuchengco School of Business, Mapua University, 1191 Pablo Ocampo Sr. Ext, Makati 1204, Philippines

**Keywords:** behavioral intention, professional athletes, sports, structural equation modeling, student athletes

## Abstract

The rise of professional careers in playing sports has been seen in the recent generation. Ranging from traditional sports to recent e-games, it can be seen that student athletes are not only considering these as motivational factors for education, but also as future career paths. This study aims to fill the knowledge gap about the factors influencing college athletes’ aspirations to play professionally. The study examines the complex web of athlete decision-making by utilizing the extended Theory of Planned Behavior (TPB) and analyzing the roles of motivational factors, self-efficacy, affective behavior, and behavioral domains. With the use of structural equation modeling, the study deciphers the intricate links, emphasizing the critical role that attitude plays in affective behavior. It emphasizes how much optimism and self-efficacy shape an athlete’s behavior and subsequent pursuit of professional careers. The current research provides a benchmark for future studies exploring student athletes’ career goals. This research adds understanding to the knowledge gap regarding the complex decision-making procedures of aspiring professional athletes. Both theoretical and practical implications are provided based on the results of the study, which stakeholders and institutions may consider for student athletes wanting to pursue a professional sports career.

## 1. Introduction

The challenges associated with pursuing a career in professional sports are increasing globally, fueled by a desire for success and escalating incomes. The gross domestic product (GDP) of professional players across the world has been discussed to be wildly different as it is measured based on the type of sport (such as basketball, volleyball, and even mobile games) and population, and success is measured based on medals and winning, affecting demographic and economic factors [1]. As expressed by Aygun et al. [2], societies within countries have capitalized on sports to gain both cultural and social development. Of note, it is evident that different countries have recognized football as a highly professional sport, followed by basketball, and then baseball, to name a few. Additionally, sports and its industry have accounted for 3% of economic activities on a global scale [2].

In Asia, the rise of professional sports careers has also been evident and recognized. GDP growth in Asia has averaged 5.5% over the past decade [3], aligning with a rising middle class with greater funds to spend on entertainment activities. Occupations within professional sports are becoming increasingly popular because of their entertainment aspects, increasing media markets, and growing popularity abroad, of which basketball has become extremely popular. Based on the study by Colorado [4], the Chinese Basketball Association’s average player salary is currently the highest in the world, at over $3 million annually. Meanwhile in the Philippines, a country with a GDP per capita beyond $4000 [3], the Philippine Basketball Association offers competitive earnings and a national following, with top players earning up to (approximately) $1 million yearly [5]. E-sports in Southeast Asia, particularly in the Philippines, have already started attracting significant sponsors and investors that create career paths for skilled players [6].

In order to succeed and have a successful professional career, players should be exceptionally talented and have an unwavering dedication to the sport due to numerous competitive players [7,8]. The term of employment is limited, and the physical and mental demands are high, but there is potential for large financial returns [8,9,10,11]. They present a chance not only to attain peak physical and athletic excellence but also leave a positive impact on the developing sports scene in this peculiar geographical area of the Philippines. There are also substantial financial benefits and even cultural benefits to pursuing a professional sports career in the Philippines, and an athlete receives great and competitive support in international events [12].

However, pursuing this route means there is no guaranteed path to success. Additionally, several significant obstacles regarding sustainability and the well-being of aspiring athletes usually raise questions. Therefore, the development, encouragement, and enhancement of professional athletes in the country have become a challenge. One of the main problems is the fierce competition for a few spots in the team. Specialization is usually needed in this intensive battle, which increases the risk of burnout and academic neglect among student athletes [13]. Furthermore, the strain of continuously delivering a perfect performance can worsen mental health issues and lead to an addiction to dangerous performance-enhancing techniques [14]. Despite the financial incentive appearing tempting, athletes often have uncertain careers and inconsistent salaries once they retire from the competition [15]. Moreover, socioeconomic status often dictates an athlete’s access to excellent training facilities, experienced coaches, and adequate financial support, creating an unfair playing field [16]. This brings up ethical issues about the distribution of opportunities equally and the potential exploitation of young athletes from low-income families. 

Measuring these challenges is still difficult, which is why there are only a few research studies on professional sports career paths (further presented in the succeeding section). In a broader sense, it is significant to consider our limited comprehension at the fundamental level of understanding the factors influencing participation and engagement in professional playing careers. It has been observed that there is a lack of greater research evidence to guide and understand players’ decisions to pursue professional careers. For instance, research conducted by Biddle et al. [17] on quantitative systematic reviews concluded that, apart from age and gender, factors that impact participation are expected to have minimal influence when examined separately. Their effectiveness may increase when they interact with other influences, although the precise details of these interactions contributing to motivation are still yet to be identified.

The current knowledge gap for pursuance intention among athletes needs to be deciphered since the establishment of sports programs in the Philippines is increasing [18,19,20], especially since this has been explained to be a game changer in the country’s economy [21,22]. In addition, professional careers among athletes have been seen to be considered by the younger generation [23]. Thus, the need for exploration among intention to instigate initial programs, assessment, and promotion by institutions could be built. By understanding the underlying factors, government, institution, and stakeholders such as coaches and sponsors could promote the career path student athletes would want to pursue.

The research of Ong et al. [24] and Hollett et al. [25] explored the relation of the TPB as a generalized approach to assessing individuals’ behavior and intentions. Therefore, their study has extended the model by incorporating external variables to have a more holistic and specific assessment rather than the TPB alone. As a reflection, this study aims to determine factors influencing collegiate players’ intention to pursue a professional playing career. Specifically, this study considers the TPB domains of perceived behavioral control, subjective norm, and attitude, including the extension variables of motivation (both extrinsic and intrinsic), as well as affective behavior and self-efficacy. This way, a coherent and holistic assessment, analyzed using structural equation modeling, can be obtained.

## 2. Related Studies and Conceptual Framework

### 2.1. Related Studies

Among related studies, young adults typically begin assessing their chosen careers during their initial years of high school [26]. However, the studies presented that young individuals are significantly affected by guidance/input from family, parents, peers, and coaches [26,27,28,29,30]; however, not everyone has the same impact on their choices. It has been presented that development, beliefs, representation, and views have affected the decision of students to become student athletes [7,31]. During this procedure, the decision is significantly influenced by perceptions of reality rather than the actual reality. Earlier studies have already speculated and supported the current standing. For example, Super’s [32] study explained that professionals in collegiate athletics and academic/student affairs should integrate career development as a guiding framework to facilitate, engage, and assist students in achieving their career goals. It was seen that, without bias, it would help make these decisions towards their career an actual reality. Martini et al. [33] observed that in today’s generation, individuals frequently make continuous decisions, reassess their past professional choices, and consistently modify their behaviors and influences. 

To assess the intention of individuals, Ajzen [34] suggested that the Theory of Planned Behavior (TPB) provides a valuable framework for understanding why behavioral domains relate to the influence to pursue a professional career. Studies applying TPB to athlete career paths have indicated the influences of peer pressure, resource availability of career targets, and parental expectations [35]. As a result, it was proposed that overcoming these challenges requires an integrated approach. Promoting engagement with different kinds of sports, maintaining players’ mental health and well-being as a priority, and then ensuring equal access to training facilities and resources are all crucial steps. Improving financial awareness among athletes and putting job transition programs into effect can also help them prepare for life beyond sports. It was added that creating a more ethical and sustainable route for gifted athletes in the professional space in Asia, including the Philippines, by understanding the challenges that come alongside the rewards is needed to enhance the development of professional athletes. 

### 2.2. Conceptual Framework

**Figure 1:**
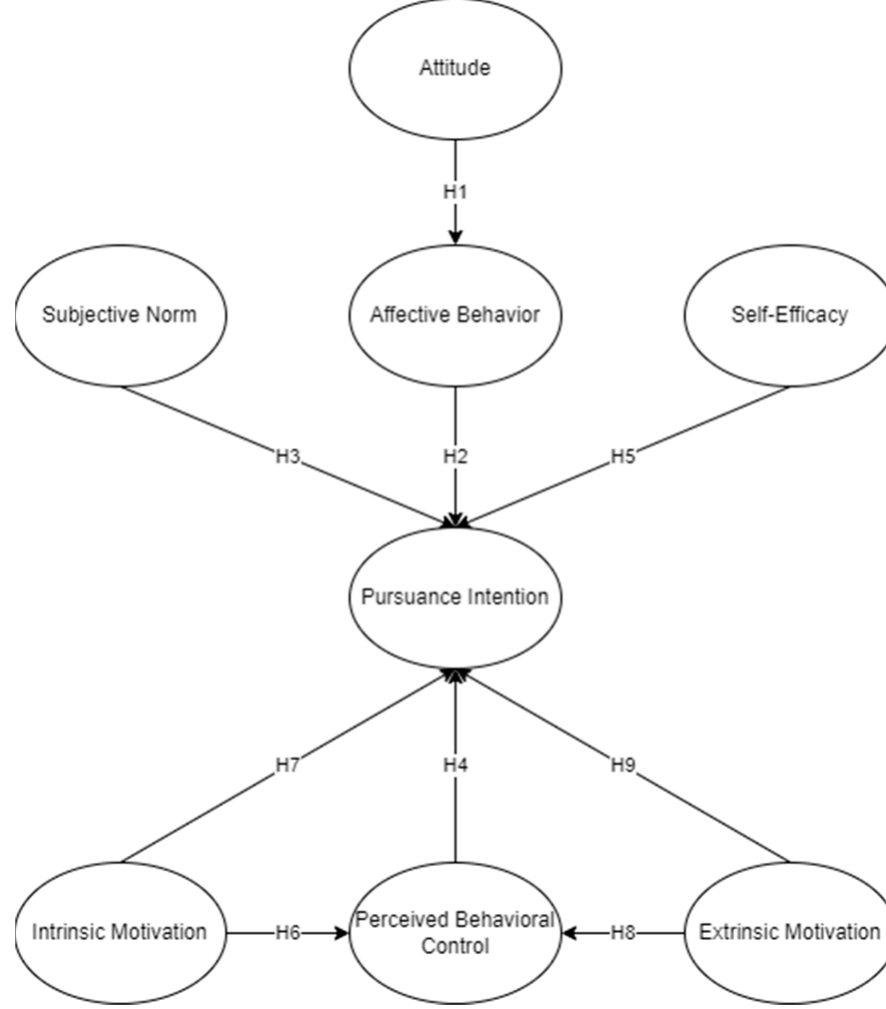
Conceptual Framework.

Figure 1 shows the factors adapted from related studies that contribute to collegiate players’ intention to pursue a professional career. A total of seven variables were hypothesized to have different effects on the players’ intention to pursue a professional career such as attitudes, subjective norms, and perceived behavioral control, alongside the extended variables of self-efficacy, affective behavior, and extrinsic and intrinsic motivation.

A previous study has already proved that attitude is measured by the emotional dimensions of both valence and arousal that are associated with the intention of players towards their career [34]. The attitude associated with most players is in terms of assessment, knowledge about their sports, beliefs, thoughts, and individual opinions regarding the downside and benefits of their actions. The actions of players also have a vital role in their motivational process because of the factors that can affect their choices that result in an intention, an affective behavior. Any behavior that involves a lot of planning and effort can be measured by the intention to adopt that certain behavior [36]. 

According to Yavuz [37], the correlation between attitudes toward sports and general life achievement shows the broad impact of sports on an individual’s behavior and achievements. Previous studies also show that negative attitudes hinder students’ performance, which results in poor performance [38,39,40]. However, in recent generations, Park et al. [41] found that the most significant factor determining behavioral intentions is attitudes towards a behavior. Izard [42] recommended the existence of 10 different human emotions, and that each emotion plays a role in the behavior of individuals and has a distinctive experiential aspect. Within these emotions, interest and excitement is identified as the foundation for pursuance intention, with joy playing a secondary role. Ong [22] also related attitudes and affective behavior, affecting pursuance intention in the academic setting. Relating to attitude, it was hypothesized that:

**H1:**  *Attitude has a significant effect on affective behavior.*

Research by Deci and Ryan [43] and Ong [22] examined motivation as a moderator in the relationship between intention and behavior, which focused on the degree to which intentions are derived from affect or identity. The study suggested that intentions rooted in personal or affective factors, as opposed to other influences, exhibit stronger predictability for behavior, aligning with the expectations of self-determination [22]. Intrinsic motivation was recognized as the example of autonomous motivation, representing the drive to engage in behavior for the interest and satisfaction derived directly from the action of the individual itself. The repetition of past performances of a behavior or habit of the individual, as assessed through self-report measures, has been identified as a moderating factor in the intention–behavior relationship [44]. Individuals can recognize the different points of autonomous and controlled motivations for their actions, and the proportionate influence of these factors is likely to determine whether individuals will persist with or discontinue the behavior in the long term [45]. Thus, it was hypothesized that:

**H2:**  *Affective behavior has an effect on pursuance intention.*

A belief that an individual or group will approve and support a particular behavior is called a subjective norm. This is shaped by an individual’s motivation to live up to the opinions of others and how they think of peer pressure from others to behave in a particular way [46]. As outlined by Ajzen and Fishbein [34], this aspect involves societal pressure affecting individual intention and is often measured by the extent of innovative compliance. Based on the findings of Putra and Purba [47], student interns’ intention to apply for jobs or other internships being offered by different companies was a predictor of subjective norms and self-efficacy. According to the study by Daudi et al. [48], students’ attitudes had the most predictive power over subjective norms and perceived behavioral control, but all three TPB domains had a significant effect on their career intentions in their respective industries. When it comes to career pursuance, the subjective norm has been proven to have a significant effect because people might be inspired to pursue jobs that are aligned with societal expectations, especially if people think that influential individuals and groups will approve of them [49]. Furthermore, the need to avoid disapproval increases the impact of subjective standards on career choices [48]. It could be posited that stronger subjective norms influence the tendency of individuals to follow career paths that fit the societal norm, even if those paths do not match with their individual preferences. Therefore, it was hypothesized that:

**H3:**  *Subjective norms have a significant effect on pursuance intention.*

In earlier studies, Walston [50] stated that perceived behavioral control (PBC) is a lot like self-efficacy, or it refers to the person’s beliefs that their behavior is under their control or not. The difference is that PBC attributes this to one’s ability to exhibit a particular behavior, while self-efficacy involves one’s ability to trust their capabilities to organize and execute the actions needed to achieve what is needed [51]. This suggests that self-efficacy has a broader scope and includes an individual’s perceived control over their complete course of action towards a specific goal, while PBC emphasizes the difficulty of behaving a certain way. 

According to the research of Jae Gu Yu and Jeong [52], PBC directly influences the choices people make to pursue a career. Based on their findings, people with higher PBC are more likely to choose a career they believe is manageable and achievable. The self-determination theory of Deci and Ryan [43] explains that individuals have a natural desire to pursue activities they consider to be autonomous and controlled. Individuals are more likely to be engaged and persistent in their professional careers when they think they have more control over important choices, which in return enhances their intention to pursue those careers. It is reasonable to believe that PBC has a significant effect on pursuance intention. Therefore, it was hypothesized that:

**H4:**  *PBC has a significant effect on pursuance intention.*

Self-efficacy, the perceived capacity of an individual to execute tasks essential for professional success or adjustment, plays a pivotal role in various domains [53]. Athletes, with a fundamental understanding of their ability to navigate different challenges and accomplish their goals, have a lean edge in having a successful career. Achieving a successful career would mean many decisions must be made. According to Cabrita et al. [54], there is a positive correlation between a higher athletic identity and increased self-efficacy levels in career decision-making, which could help student athletes plan their career. However, Lally and Kerr [55] shed light on the evolving nature of a student athlete’s identity, emphasizing its influence on career decisions during college and post-college life. Moreover, Heazelwood and Burke [56] highlighted a strong correlation between self-efficacy and the performance of triathletes. Their study indicated that athletes with higher levels of self-efficacy consistently outperformed their counterparts with lower self-efficacy levels. This finding underscored the significance of psychological attributes—such as resilience and confidence—in augmenting athletic achievements, supplementing the importance of physical training in athletic success. Furthermore, self-efficacy is one important motivational process influenced by human factors and the environment. It drives out motivational outcomes like effort, perseverance, achievement, and choice [57]. It profoundly impacts decision-making, goal-setting, and sustained perseverance amidst challenges, not only within athletic pursuits, but also across many domains. Beyond individual accomplishments, self-efficacy also shapes group dynamics and collective efficacy within teams. According to the study by Cassidy [58], motivation and having encouraging surroundings can boost motivation and resilience, which can easily spread to fellow athletes. It was explained that increasing players’ self-efficacy can be an effective method to improve individual and team success. Therefore, it was hypothesized that:

**H5:**  *Self-efficacy has a significant effect on pursuance intention.*

Motivation in its simplest form can be described as the capacity to initiate and sustain a task. According to the study by Taylor [59], motivation serves as the fundamental underpinning of athletic endeavor and achievement. Yukhymenko–Lescroart [60] stated that motivation is a crucial factor in a person’s determination to achieve long-term goals. They found that there is a positive relationship between athletic motivation and career adaptability, considering all the traits and skills necessary to succeed as an athlete. The research highlighted that student athletes, who would often need to manage deals involving their name and image, were particularly good at career adaptability. This ability was connected to many student athletes showing that they are motivated to pursue sports professionally [61]. 

On the contrary, earlier studies also suggested that athletes are also driven by intrinsic motivation [62,63]. According to Deci and Ryan [43], PBC entails participating in activities purely for the interest and enjoyment they provide. Intrinsic motivation is exemplified by behaviors like play, exploration, and activities driven by curiosity, as they are not affected by external rewards or pressures. Rather, these activities inherently offer their own satisfactions and joys, emphasizing the connection between intrinsic motivation and the individual’s perceived ability to control and engage in such behaviors. Therefore, PBC has a significant effect on intrinsic motivation. It was presented that athletes make their sport their identity and strive to improve their sports skills. Some also considered their sport a serious leisure, an activity considered as work but a casual leisure with benefits. It was seen that two distinct categories of intrinsic motivation emerge in the context of athletes [64]. The first pertains to an athlete’s internal drive and personal interest in their chosen sport, reflecting their inherent passion and dedication. The second form of intrinsic motivation centers around athletes being motivated by the success and aspirations of their peers, coaches, and teammates who share the same goal of achieving professional excellence in their respective sports. Athletes can be motivated to achieve their peak performance and make significant improvements to both individual and team success by building and maintaining intrinsic motivation through internal and external factors. Thus, this study hypothesized that:

**H6:**  *Intrinsic motivation has a significant effect on PBC.*

**H7:**  *Intrinsic motivation has a significant effect on pursuance intention.*

The self-determination theory of Deci and Ryan [43] explains that individuals have a natural desire to pursue activities they consider to be autonomous and controlled. Individuals are more likely to be engaged and persistent in their professional careers when they think they have more control over important choices, which in return enhances their intention to pursue those careers. It is reasonable to believe PBC significantly affects extrinsic motivation. 

It was argued that most athletes are driven by extrinsic motivation, which includes money, fame, and recognition, to pursue a professional playing career [65]. Many male athletes from the Pacific region perceive professional playing careers as a path to better social mobility. The significant financial rewards that can be earned as a professional athlete can improve status and reputation [66,67]. In e-sports, most young gamers are considering pursuing a professional career due to earnings that include cash prizes, team salaries, viewers from streaming, and sponsorship money [68,69]. Other than financial gain, Wylleman and Reints [70] also suggested that recognition from parents is crucial and encourages athletes to stay motivated in playing their sport. It was also emphasized in a study that the proactive roles of parents contribute to student athletes’ motivation to play and develop their skills [71]. Between intrinsic and extrinsic motivation, perceived behavioral control serves as a mediator. It can convert externally motivated behaviors into independent engagement by encouraging competence and autonomy. This knowledge has significant implications for supporting constructive behavioral change. When combined with extrinsic motivators, programs that aim to increase perceived control are likely to be more successful in bringing forth long-lasting change than those that can only rely on incentives or pressure from outside sources [72]. Therefore, it was hypothesized that:

**H8:**  *Extrinsic motivation has a significant effect on PBC.*

**H9:**  *Extrinsic motivation has a significant effect on pursuance intention.*

## 3. Methodology

### 3.1. Participants 

A total of 351 respondents answered the survey. This study considered respondents who are collegiate athletes in the Philippines with these respective sports: 3 × 3 basketball, athletics, badminton, baseball, basketball (men and women), beach volleyball, Call of Duty: Mobile, cheerleading, chess, fencing, football (men and women), Judo, lawn tennis, League of Legends, Mobile Legends, softball, street dance, swimming, table tennis, Taekwondo, Valorant, and volleyball (men and women). The survey was conducted via Google Forms and administered online from November 2023 to January 2024 to assess the insights of the different collegiate athletes in the Philippines. 

Table 1 shows the descriptive statistics of the target demographic profiles of college student athletes from the Philippines. A total of 63.2% are male, and 36.8% are female with ages ranging from below 18 years old (3.1%), 18–25 years old (88%), and above 25 years old (8.8%). The type of school of each respondent was also asked about, between public (16.2%) and private (83.8%). Participants’ monthly allowance/income was also considered as less than 10,000 PHP (25.6%), 10,001–20,000 PHP (46.4%), 20,001–30,000 PHP (20.8%), and 30,001 and above (7.1%). 

Participants’ duration of engagement in their respective sports was also examined, with 6.3% playing for less than a year, 43.9% for 1–2 years, 16% for 3–4 years, and 33.9% for five years or more. Furthermore, the survey included an inquiry into the specific sports undertaken by respondents, revealing the following distribution: 1.1% for 3 × 3 basketball, 2.8% for athletics, 9.4% for badminton, 5.4% for baseball, 28.5% for basketball (men and women), 1.7% for beach volleyball, 1.1% for Call of Duty: Mobile, 0.9% for cheerleading, 2.8% for chess, 0.9% for fencing, 9.1% for football (men and women), 0.3% for Judo, 0.6% for lawn tennis, 2% for League of Legends, 1.4% for Mobile Legends, 2% for softball, 1.7% for street dance, 2.3% for swimming, 6% for table tennis, 1.4% for Taekwondo, 5.4% for Valorant, and 13.1% for volleyball (men and women).

### 3.2. Measure Items 

The questionnaire was divided into 7 variables: (1) perceived behavioral control, (2) subjective norms, (3) attitude, (4) extrinsic motivation, (5) intrinsic motivation, (6) affective behavior, and (7) self-efficacy, which were adapted from various research works with similar individual frameworks [42,43,44,45,46,47,48,49,50,51,52,53,54,55,56,57,58,59,60,61,62,63,64,65,66,67,68,69,70,71,72,73,74]. The survey was employed using a 5-point Likert scale with “1—Strongly Disagree” to “5—Strongly Agree”. Presented in Appendix A (Table A1) are the measure items considered for assessment in this study. Prior to dissemination, the Ethics Committee reviewed the measure items and the final questionnaire, which was subjected for a preliminary survey test. A total of 50 respondents were considered for the assessment, structure of the survey, and clarity. An overall Cronbach’s alpha value was obtained to be 0.824, which was set to be acceptable from the multivariate analysis discussion by Hair [75].

### 3.3. Structural Equation Modeling 

Structural equation modeling (SEM) is a highly utilized multivariate statistical technique that involves elements from factor and path analysis to investigate intricate interactions between observed and unobserved variables within a theoretical framework [76]. By simultaneously modeling direct and indirect effects, correcting for measurement errors, and assessing the model’s fit to the data, this enables researchers to test the validity of ideas. According to Dash and Paul [77], they proved that both covariance-based SEM (CB-SEM) and partial least squares SEM (PLS-SEM) could be utilized when base frameworks are established. PLS-SEM is considered as a newer version of CB-SEM, which is advantageous since it considers a general approach and is fully developed [78]. Additionally, factor analysis is the focus of CB-SEM, while PLS-SEM is more holistic—considering the total variance of the model in accordance with the response made. The common, specific, and error variance are inclusive in the calculation with PLS-SEM.

Moreover, the representation of the linear-based combination of the indicators presents a better analysis. The rule-of-thumb discussion made by Hair et al. [79] explained that the number of respondents should be greater for the analysis to be suitable, but this was shown to have no significance by other studies [78]. This is because recent findings have indicated that smaller sample sizes could already be tested with PLS-SEM [77,78]. Other than that, studies have already established the use of PLS-SEM, especially in the social science field due to its sensitivity and holistic measurement approach. The only distinction made was for CB-SEM to be used when frameworks are only established and tested for specific cases [77]. Since the current study utilized an extended TPB model, PLS-SEM was considered. The flexibility of SEM presents distinct advantages in the analysis of complex causal structures, especially if analyzing latent constructs (such as affective behavior, and attitude) that are not easily quantified. However, due to its complexity, model specification, estimation, and evaluation, procedures should be thoughtfully considered [80]. Therefore, the current study utilized the Smart-PLS v3 for the analysis performed. 

## 4. Results

### 4.1. The Result of Initial SEM

**Figure 2:**
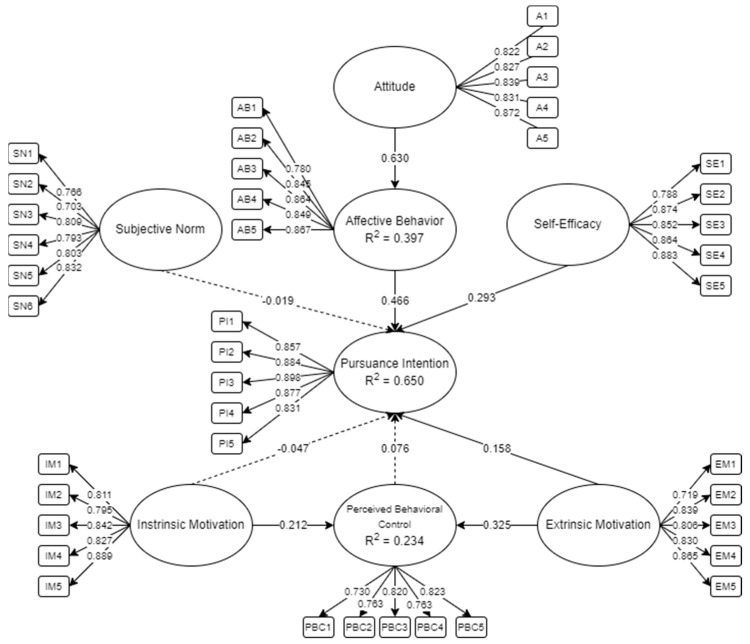
Initial SEM model.

The initial SEM for determining the factors influencing collegiate players’ intention to pursue a professional playing career is presented in Figure 2. The model consists of 8 latent and 41 indicators. Following the suggestion of related studies [75,77,80], factor loadings of less than 0.70 should be removed as insignificant measures of the latent variable. In accordance, relationships with *p*-values greater than 0.05 are insignificant [75]. Therefore, these could be removed to enhance the model fit of the SEM. Presented in broken lines are the relationships with higher *p*-values than the threshold set.

Table 2 shows the descriptive statistics of the response, alongside the initial and final models. The measure items had factor loadings greater than 0.70. Following the suggestion of Dash and Paul [77], all measure items would be deemed acceptable when the threshold is reached. In accordance, it could be seen that the final factor loading of SN was removed since the relationship was considered to be insignificant.

Subsequently, to evaluate convergent validity, the average variance extracted (AVE) and composite reliability (CR) were calculated, alongside Cronbach’s alpha. Table 3 indicates that all constructs in the model meet the necessary thresholds for validity and reliability [78,79,80].

To demonstrate, discriminant validity was taken into consideration utilizing the heterotrait–monotrait correlation ratio (HTMT) and the Fornell–Larcker criterion (FLC) to evaluate the measurement model and the correlation between each variable [81]. As outlined by Hair [75], the FLC should have diagonal values greater than the respective horizontal and vertical values for it to be deemed acceptable. According to Kline [76], discriminant validity is established by an HTMT below 0.85. As shown in Table 4 and Table 5, the values fall within the set ranges, indicating satisfactory reliability and discriminant validity. 

### 4.2. Model Fit Analysis

An assessment of model fit was conducted to prove the validity of the proposed model. Table 6 demonstrates that all parameter estimates were higher than the minimal threshold value as suggested, proving the acceptability of the suggested model. 

### 4.3. The Result of the Final SEM

The result of the final SEM underscores several pivotal factors shaping an individual’s intention to pursue a professional career, as summarized in Table 7. It could be posited that attitude and affective behavior emerged as the most influential (β = 0.630, *p* < 0.001), following which is affective behavior and pursuance intention (β = 0.474, *p* < 0.001). However, extrinsic motivation and PBC had a significant but somewhat smaller effect (β = 0.323, *p* < 0.001) compared to extrinsic motivation and pursuance intention (β = 0.148, *p* < 0.001). Moreover, self-efficacy and pursuance intention also stands out as a significant factor (β = 0.293, *p* < 0.001). Additionally, intrinsic motivations positively contribute to PBC (β = 0.213, *p* = 0.006), and in contrast, perceived behavioral control (β = 0.079, *p* > 0.05) and subjective norms (β = −0.019, *p* > 0.05) showed no significant effect on pursuance intention. Lastly, an interesting finding surfaced with intrinsic motivation and pursuance intention, displaying an unexpected negative nonsignificant relationship (β = −0.047, *p* > 0.05). 

**Figure 3:**
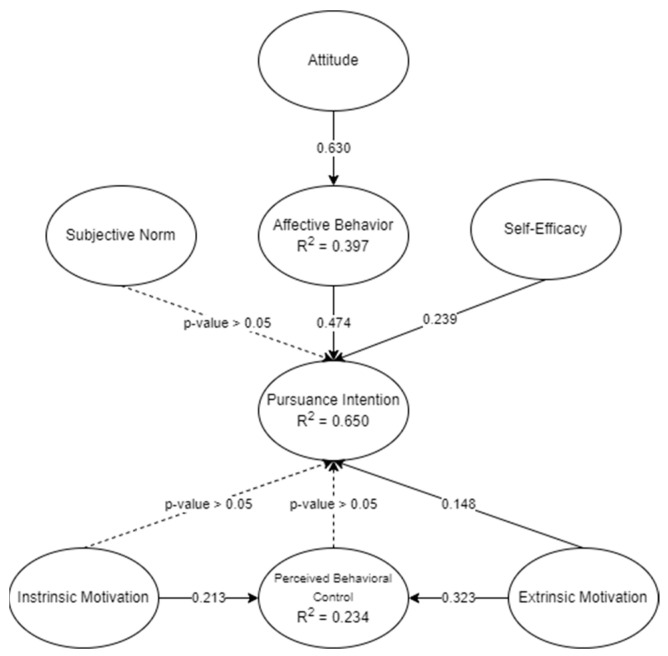
Final SEM model.

The depicted SEM model is presented in Figure 3, wherein the beta coefficients and R^2^ values were calculated to evaluate the hypothesis model. The model attributes 64.5% of the variation to pursuance intention, 39.7% to affective behavior, and 23.4% to behavioral control. This suggests that the model adequately explains or predicts the perceived pursuance intention for the professional playing career of an individual with values greater than the 20% threshold [80].

The covariances of the exogenous latent variables were calculated. As presented in Table 8, the covariances are all significant (*p*-value < 0.001). Relating to Mir et al. [85], they explained how the estimates promote the positive effect between relationships. Since all the exogenous variables are related to a positive estimate and significant output, it could be posited that they provide relevance to the measurement of the study. From the results, greater self-efficacy would be seen with higher attitude—leading to greater pursuance intention. However, lesser pursuance intention would be seen when social norms are considered even if attitude is positive. Similar effects are seen on intrinsic motivation and pursuance intention but could be posited to be mediated by perceived behavioral control to positively influence pursuance intention. The direct effect, however, would not have any significant effects.

In contrast, attitude, self-efficacy, and intrinsic motivation have a positive effect on extrinsic motivation and pursuance intention. This may be directly or indirectly mediated by perceived behavioral control, and the positive influence of extrinsic motivation would provide positive pursuance intention.

To further provide insights, a clustering test was conducted to ensure that the measure items, in relation to the response, presented significant differences. In this analysis, cross-cluster influences were checked to distinguish any items influencing other variables. Hierarchical clustering was conducted to ensure the validity of the output. Using hierarchical clustering with Ward’s method, three clusters would not present any significant differences in the reduced error. Therefore, the analysis of variance (ANOVA) was conducted to test for any significant difference among the responses. Presented in Appendix B are the ANOVA outputs for the summarized results (both between and within groups), indicating that there are significant differences among the response and measure items. This means that there were no errors between measurements; therefore, the SEM analysis conducted is acceptable. Moreover, presented in Appendix C are the descriptive outputs from the three-cluster results.

## 5. Discussion

From the results, it was seen that attitude had the most direct and dramatic impact on affective behavior (β = 0.630, *p* < 0.001). This observation shows that respondents, who are driven by a positive mindset and confidence in the result of their hard work are more likely to exhibit positive affective behavior. It could also be posited that respondents draw motivation from their long-term goals. According to Singh and Gupta [86], commitment refers to being devoted to a cause or objective, while an intention is a purposeful aim or plan. It could be posited that a commitment towards the improvement of their individual skills and keeping their focus on maintaining a healthy and balanced lifestyle as athletes, giving them a positive approach to the game, is significant. These factors collectively contribute to the observed significant relationship between attitude and affective behavior, which leads to the pursuance intention of an individual. 

Second, it was seen that affective behavior was considered as significant in collegiate players’ intention to pursue a professional playing career (β = 0.474, *p* < 0.001). This finding underscores that respondents’ experience heightened excitement envisioning themselves as professional players, exhibiting a strong eagerness to embark on a career in professional playing. The respondents expressed a profound passion for dedicating their lives to the pursuit of a professional player’s career, eagerly anticipating the challenges and growth opportunities associated with it. It could be seen that being a professional athlete would be extremely fulfilling for them. According to Batucan et al. [87], it was claimed that a sense of fulfillment and engagement in players’ respective sports fuels the desire for continuous development and drives them to seek the higher-level competition commonly found in college athletics. Moreover, positive affective behavior plays a crucial role in fostering resilience in the face of adversity [88]. Athletes inevitably encounter setbacks and challenges, but the experience of positive emotions such as optimism, confidence, and gratitude enable them to rebound and sustain their commitment to their objectives [89]. This resilience then becomes particularly essential in navigating the rigorous landscape of collegiate athletics, where athletes contend with academic pressures, intense competition, and potential injuries [90].

Third, extrinsic motivation was proven to significantly affect PBC (β = 0.323, *p* < 0.001). Notably, factors such as financial gains and desire for fame are crucial in motivating dedication to the goal of a professional playing career. Respondents expressed that those external incentives, including the goals set by their coaches and the desire to please both mentors and teammates, contribute significantly to their sustained motivation. Extrinsic motivation describes actions fueled by external factors, such as higher standards for job demands and the reputation that comes with being a professional [91]. The potential of developing a significant social media following alongside the opportunity of accolades and awards drives the players to keep going. Previous research by Zhu and Liu [92] emphasized that from the perspective of extrinsic motivation, competition influences a person’s pursuance intention and serves as an established learning process with predetermined objectives. 

Fourth, it was seen that self-efficacy also significantly influenced pursuance intention (β = 0.293, *p* < 0.001). The importance of self-efficacy for sustaining consistent performance in their sports was emphasized by the respondents. They showed a strong belief in their abilities to develop and acquire the necessary skills needed for a professional athlete. Confidence in handling pressure and meeting the demands of such a career was highlighted, displaying robust self-assurance in managing the challenges associated with professional play. Furthermore, it could be posited that respondents conveyed confidence in their ability to make critical decisions concerning their professional playing career, underscoring a sense of autonomy and decisiveness [93]. Additionally, a steadfast belief in overcoming the setbacks and challenges associated with pursuing a professional career was emphasized by August [94], reflecting resilience and determination in the face of obstacles. Another factor influencing undergraduate students’ career intentions is self-efficacy, which is a person’s belief in their capacity to succeed in circumstances, complete a task, and meet predetermined performance objectives.

Fifth, intrinsic motivation proved that there is a connection to PBC (β = 0.213, *p* ≤ 0.006). According to the results, the respondents’ strong commitment in pursuing a professional playing career is fueled by a genuine passion and love for the sport. They do derive joy and satisfaction from both training sessions and skill enhancement activities. Based on the study of Deci and Ryan [43], intrinsic motivation represents the most internally driven and independent type of motivation. It drives individuals to participate in activities purely for the pleasure and satisfaction derived directly from the activities themselves that affect their behavior. In essence, intrinsically motivated individuals engage in activities for the sheer enjoyment, interest, and fulfillment they provide [95]. The satisfaction of achieving personal goals significantly influences dedication and effort in the sport. Overall, the passion for their current sport is a driving force in their pursuit for a professional career [96]. Moreover, Deci and Ryan [43] explained that players desire to see themselves as actively choosing to engage in behaviors by their own free will. The accomplishment of the need for proficiency occurs when individuals sense that they are acquiring skills and achieving mastery in the activities they undertake which manifest through their behavior [96]. 

Lastly, extrinsic motivation proved to have the lowest significance in collegiate players’ intention to pursue a professional playing career (β = 0.148, *p* < 0.001). Extrinsic motivation, encompassing external rewards and incentives, emerged as a crucial factor shaping athletes’ intentions to pursue a professional career. Tangible rewards like scholarships, athletic grants, and professional opportunities prove to be compelling motivators for athletes entering collegiate careers—which are widely considered in the Philippines due to the expense of education [97]. These incentives not only provide a sense of security and validation but also hold appeal for athletes from underprivileged backgrounds, for whom college represents a pathway to financial stability and upward mobility [98]. Athletes who secure scholarships or professional contracts are more likely to intensify their efforts in training and skill improvement, thereby enhancing their chances of success [99]. However, it is not a guarantee that the support they receive from the school will be fair to every individual, which could thereby negatively affect pursuance intention [97]. 

Furthermore, social recognition and accolades stood out as potent motivators for athletes seeking external validation and affirmation. The prestige associated with participating in a renowned college program, achieving championship victories, or receiving awards contributes significantly to an athlete’s social standing and self-esteem [100]. Additionally, extrinsic motivation plays a crucial role in sustaining engagement during challenging collegiate-level training routines [101]. The initial drive required to navigate intense training and competition schedules often stems from the pressure to consistently perform at a prominent level and secure continuous recognition.

Although the respondents acknowledged potential financial limitations, the data surprisingly showed that PBC did not significantly influence their aspirations to become professional athletes (β = 0.076, *p* = 0.100). This suggests that their internal drive and confidence in their abilities outweighed concerns about external obstacles. If the respondents possessed strong extrinsic motivation and self-efficacy, they might have already factored in potential challenges and believed they could overcome them, regardless of perceived limitations [71]. Based on the study of Ru et al. [102], PBC is a critical determinant of individuals’ pursuance intention. Thus, coaches, family, friends, and mentors should think about how to make it easier for individuals to seek motivation and inspiration to achieve their goals. 

More so, it was seen that the subjective norm was proven to have no significant effect on pursuance intention (β = −0.019, *p* = 0.712). According to the respondents, professional experts and coaches/mentors significantly impacted their decision to pursue a career as professional athletes. However, it could be deduced that they felt no pressure to pursue this goal. While the influence of public perception was acknowledged, family influence was cited as a key factor in their decision-making. Based on the study of Howard et al. [103], it was anticipated that parental behaviors and family gestures that enrich supportive and encouraging environments for children’s independent expression and personal growth would be associated with stronger intrinsic motivation and improved achievements, rather than an effect on subjective norms. Respondents additionally stated that being inspired to pursue a professional playing career by the accomplishments of professional players was an important driver. In this context, Wylleman et al. [70] proved that achieving success in an elite sports career demanded significant commitment during the earlier years. During this time, athletes acquire the requisite skills and experience needed to compete at the highest levels and transition their sports involvement into a profession that the players’ professional players have already experienced. 

If the respondents had strong personal values related to pursuing their dreams, or a high value on individual decision-making, they might have been less influenced by social expectations compared to others [104]. The strength of social pressure around the career path in question can influence the impact of subjective norms. If the respondents’ social groups were diverse in their opinions about professional playing careers, or if the career is less conventional, then subjective norms might have had influence [105]. In such cases, individual values, and the variety of perspectives within their social circles, could have played a more substantial role in shaping their pursuance intentions. 

Lastly, it was discovered that intrinsic motivation had no significant effect on pursuance intention (β = −0.047, *p* = 0.489). Respondents clarified that their innate love and passion for their sport remains the primary source of motivation for their dedication in pursuing a professional playing career. They place significant value on feeling content and happy while learning new skills, which suggests that their motivation comes from their inherent enjoyment of the activity. Remarkably, respondents claimed that their motivation is derived only from their passion for the sport and is unaffected by other factors. The pleasure of reaching their own goals was said to have a major impact on the dedication and effort put into their respective sports [106]. This finding contradicts the study of Deci and Ryan [43], who proved that athletes who actively seek intrinsic satisfaction from the physicality of their sport, the development of skills, and the strategic challenges involved demonstrate greater resilience in the face of adversity. As expressed by their other study [107], it was believed that intrinsic motivation plays a primary role in most individuals’ learning throughout their lives, rather than externally imposed learning and instruction. Patall et al. [108] supported that the general intention to pursue something positively impacts intrinsic motivation when individuals are free to choose. Additionally, Patall et al. [109] demonstrated that teachers can promote autonomy by considering students’ interests, which tends to lead students to perceive them as more competent [110]. 

### 5.1. Theoretical Implications

This study provided an extended framework for evaluating athletes’ career aspirations, and aspects of the TPB with internal (motivation, self-efficacy) and external factors (subjective norms, perceived control). This whole approach has excellent value for researchers, organizations, and educators in sports psychology, behavior, and career counseling. This study could help future athletes make well-informed decisions about their pursuance intention for future careers and academics. From the established framework, it could be posited that a positive and holistic outcome was presented. This, therefore, could be capitalized on by stakeholders, even future researchers, when analyzing future and pursuance intention among student athletes and related fields. The framework utilized in this study holds relevance beyond the domain of sports that could be adapted and extended to measure the individuals’ future intentions in various fields, even outside professional careers. Furthermore, the methodology and findings serve as a basis for evaluating individuals’ intentions in a diverse behavioral domain.

### 5.2. Practical and Managerial Implications

The results of this study could have the possibility to establish a comprehensive approach for assessing individual behavior with respect to their intentions, offering universities valuable insights for fostering motivation among individuals who engage in sports, games, and other activities. In relation to the output, coaches and mentors who recognize how affective behavior affects pursuance intention can help develop a resilient and optimistic mindset that could help athletes overcome challenges in both sports and academics. As per Van Raalte et al. [111], being a student athlete constitutes a distinct career path during one’s college years, involving high academic standards alongside showcasing athletic abilities. The intersection of these domains, examined by researchers in both academia and sports, amplifies the significance of life–career success for present-day student athletes that gives the individual a positive perspective on pursuing a professional career. These findings can help managers and stakeholders in different educational institutions implement career counseling programs that consider the complex interactions between internal and external variables. Acknowledging the importance of extrinsic motivation—which includes monetary rewards and social recognition—allows educational institutions to tailor their support programs to meet the unique needs of student athletes. Furthermore, recognizing the role that self-efficacy plays in determining career plans highlights the value of skill-building programs and mentoring services designed to increase athletes’ self-assurance in managing the challenges of a professional playing career, which could be capitalized on by institutions with sports programs trying to hone professional athletes. It was examined by Kasser [112] that individual’s prioritize intrinsic goals over extrinsic ones, and that framing goals intrinsically could potentially boost the quantity rather than the quality of student athlete motivation. In addition, understanding the limited impact of PBC on career goals means programs must concentrate on enhancing self-efficacy and internal drive instead of tackling external hindrances. 

### 5.3. Limitations and Future Direction

This study still considers several limitations despite the notable findings and contributions from the results and methodological standpoint. First, this study focused only on Philippine collegiate players, which athletes in various countries or cultural backgrounds would not be able to directly utilize. Many variables, like the popularity of sports, the structures of professional leagues, and socioeconomic situations, can differ greatly and hence change the relative importance of several factors for career goals. This could therefore be a suggested consideration for future research. The indicated factors may be added as an extension or mediators affecting pursuance intention. Individual differences in personality, family support, or resource availability, for instance, can exert a complex impact on career intentions by affecting the interaction between variables like PBC and subjective norms, which this study did not cover. A more comprehensive model that considers these interactions might give aspiring professional athletes a deeper grasp of how they make decisions. Also, the study focused on the collegiate athletes’ plans to pursue professional careers as a benchmark, without digging further into the athletes’ actual career outcomes. When navigating the student athlete experience, athletes and support personnel might find it helpful to consider factors including talent, injury rates, and career management abilities that have been linked to successful transfers from collegiate to professional sports. Since this study focused on behavioral intention as a benchmark, future research may opt to consider the covariance of variables and response to uncover specifications set from the response of the student athletes. Clustering, covariance analyses, and individual specifications of the measure items and variables could be considered by future work alongside other machine-learning algorithms to support and provide other findings to further explore the benchmark study created.

## 6. Conclusions

Given the limited existing knowledge regarding the factors influencing collegiate players’ intentions to pursue a professional playing career, this study thoroughly analyzed several external and internal variables to narrow the gap. The study analyzed the intricate relationships between motivation, self-efficacy, subjective norms, and perceived behavioral control to shed light on the complexities of athlete decision-making. The extended Theory of Planned Behavior (TPB) served as the theoretical framework for this study. The study’s methodological robustness is strengthened by strict validation processes, such as convergent validity and discriminant validity tests. 

To grasp the complex relationship between these variables and the factor influencing athletes’ intents, the research utilized structural equation modeling, or (SEM). The findings emphasized the significance of attitude for affective behavior, shedding light on the profound impact of a positive mindset and confidence on athletes’ behaviors and subsequent pursuit of professional careers. Moreover, affective behavior, self-efficacy, and extrinsic motivation were also discovered to have a favorable impact on the respondents’ intention to pursue a career, while extrinsic and intrinsic motivation were found to significantly affect perceived behavioral control. 

The paper outlines the practical consequences for career counselors, educational institutions, and sports organizations. It advocates for customized support systems and interventions that are based on a thorough understanding of the elements that impact performance. It is recommended that mentors and coaches use their understanding of affective behavior to help athletes foster resilience and optimism. Educational institutions can refine their support programs to accommodate diverse motivational profiles. While acknowledging limitations, such as the study’s focus on Philippine collegiate players, the research contributes a robust foundation for future investigations into cross-cultural variations in athletes’ career aspirations. 

## Figures and Tables

**Table 1:** Demographic Profile.

Characteristics	Category	N	%
Sex	Male	222	63.2%
	Female	129	36.8%
Age	Below 18 years old	11	3.1%
	18–25 years old	309	88%
	Above 25 years old	31	8.8%
Type of School/University	Private	294	83.8%
	Public	57	16.2%
Monthly Income/Allowance	Less than 10,000 PHP	90	25.6%
	10,001–20,000 PHP	163	46.4%
	20,001–30,000 PHP	73	20.8%
	30,001 and above	25	7.1%
How long have you been playing your sport?	Less than a year	22	6.3%
	1–2 years	154	43.9%
	3–4 years	56	16%
	Five (5) years and more	119	33.9%
What is your current sport?	3 × 3 Basketball	4	1.1%
	Athletics	10	2.8%
	Badminton	33	9.4%
	Baseball	19	5.4%
	Basketball (Men and Women)	100	28.5%
	Beach Volleyball	6	1.7%
	Call of Duty: Mobile	4	1.1%
	Cheerleading	3	0.9%
	Chess	10	2.8%
	Fencing	3	0.9%
	Football (Men and Women)	32	9.1%
	Judo	1	0.3%
	Lawn Tennis	2	0.6%
	League of Legends	7	2%
	Mobile Legends	5	1.4%
	Softball	7	2%
	Street Dance	6	1.7%
	Swimming	8	2.3%
	Table Tennis	21	6%
	Taekwondo	5	1.4%
	Valorant	19	5.4%
	Volleyball (Men and Women)	46	13.1%

**Table 2:** Statistical analysis of indicators.

Variables	Item	Mean	SD	Factor Loading	
				Initial	Final
Perceived Behavioral Control	PBC1	4.034	1.009	0.730	0.726
	PBC2	4.066	0.978	0.763	0.767
	PBC3	4.031	1.063	0.820	0.816
	PBC4	3.989	1.018	0.763	0.768
	PBC5	4.077	1.128	0.823	0.824
Subjective Norm	SN1	3.952	1.120	0.766	-
	SN2	3.889	1.151	0.703	-
	SN3	3.966	1.101	0.809	-
	SN4	3.809	1.231	0.793	-
	SN5	3.806	1.253	0.803	-
	SN6	4.091	1.129	0.832	-
Affective Behavior	AB1	4.151	0.991	0.780	0.780
	AB2	3.991	1.123	0.845	0.845
	AB3	4.003	1.144	0.864	0.863
	AB4	4.006	1.075	0.849	0.849
	AB5	4.228	1.073	0.867	0.867
Extrinsic Motivation	EM1	3.718	1.178	0.719	0.719
	EM2	3.934	1.139	0.839	0.839
	EM3	3.957	1.123	0.806	0.806
	EM4	3.966	1.132	0.830	0.830
	EM5	3.792	1.318	0.865	0.865
Intrinsic Motivation	IM1	4.191	0.973	0.811	0.813
	IM2	4.219	0.996	0.795	0.793
	IM3	4.134	1.100	0.842	0.840
	IM4	4.111	1.000	0.827	0.830
	IM5	4.188	1.159	0.889	0.887
Self-Efficacy	SE1	4.077	1.006	0.788	0.788
	SE2	4.077	1.069	0.874	0.874
	SE3	4.017	1.057	0.852	0.852
	SE4	4.017	1.117	0.864	0.864
	SE5	4.123	1.144	0.883	0.883
Attitude	A1	4.202	0.925	0.822	0.822
	A2	4.248	0.936	0.827	0.827
	A3	4.214	0.962	0.839	0.839
	A4	4.219	1.016	0.831	0.831
	A5	4.333	1.083	0.872	0.872
Pursuance Intention	PI1	3.897	1.158	0.857	0.856
	PI2	3.932	1.192	0.884	0.884
	PI3	3.903	1.211	0.898	0.897
	PI4	3.954	1.181	0.877	0.877
	PI5	4.231	1.187	0.831	0.832

**Table 3:** Convergent Validity.

Variables	Cronbach’s Alpha	Composite Reliability	Average Variance Extracted (AVE)
Attitude	0.895	0.922	0.703
Affective Behavior	0.897	0.924	0.708
Extrinsic Motivation	0.872	0.907	0.662
Intrinsic Motivation	0.890	0.919	0.694
Pursuance Intention	0.919	0.939	0.756
Perceived Behavioral Control	0.840	0.886	0.610
Self-Efficacy	0.906	0.930	0.727

**Table 4:** Fornell-Lacker Criterion.

Variable	A	AB	EX	IM	PI	PBC	SE
Attitude	0.839						
Affective Behavior	0.630	0.842					
Extrinsic Motivation	0.516	0.609	0.813				
Intrinsic Motivation	0.723	0.684	0.607	0.833			
Pursuance Intention	0.595	0.752	0.604	0.583	0.87		
Perceived Behavioral Control	0.477	0.593	0.453	0.410	0.554	0.781	
Self-Efficacy	0.687	0.640	0.572	0.660	0.681	0.551	0.853

**Table 5:** Heterotrait–Monotrait Ratio.

Variable	A	AB	EX	IM	PI	PBC	SE
Attitude							
Affective Behavior	0.703						
Extrinsic Motivation	0.578	0.676					
Intrinsic Motivation	0.812	0.765	0.680				
Pursuance Intention	0.654	0.824	0.667	0.640			
Perceived Behavioral Control	0.540	0.677	0.517	0.464	0.622		
Self-Efficacy	0.763	0.709	0.634	0.737	0.745	0.626	

**Table 6:** Model Fit.

Goodness of Fit Measures of the SEM	Parameter Estimates	Minimum Cutoff	Recommended by
SRMR	0.056	<0.08	[82]
Chi-Squared	1.633	<5.00	[83]
NFI	0.928	>0.90	[84]

**Table 7:** Summarized Results.

Hypothesis	Relationship	Beta	*p*-Value	Decision
1	Attitude → Affective Behavior	0.630	<0.001	Accept
2	Affective Behavior → Pursuance Intention	0.474	<0.001	Accept
3	Subjective Norm → Pursuance Intention	−0.019	0.712	Reject
4	PBC → Pursuance Intention	0.079	0.100	Reject
5	Self-Efficacy → Pursuance Intention	0.293	<0.001	Accept
6	Intrinsic Motivation → PBC	0.213	0.006	Accept
7	Intrinsic Motivation → Pursuance Intention	−0.047	0.489	Reject
8	Extrinsic Motivation → PBC	0.323	<0.001	Accept
9	Extrinsic Motivation → Pursuance Intention	0.418	0.011	Accept

**Table 8:** Covariance Test Output.

Relationship	Estimate	S.E.	C.R.	*p*
Self-Efficacy ↔ Attitude	0.410	0.046	8.837	<0.001
Subjective Norm ↔ Attitude	0.385	0.049	7.876	<0.001
Extrinsic Motivation ↔ Attitude	0.325	0.044	7.340	<0.001
Intrinsic Motivation ↔ Attitude	0.585	0.060	9.680	<0.001
Subjective Norm ↔ Self-Efficacy	0.438	0.054	8.169	<0.001
Subjective Norm ↔ Intrinsic Motivation	0.517	0.065	7.921	<0.001
Subjective Norm ↔ Extrinsic Motivation	0.440	0.058	7.598	<0.001
Intrinsic Motivation ↔ Self-Efficacy	0.536	0.060	8.912	<0.001
Extrinsic Motivation ↔ Self-Efficacy	0.351	0.048	7.372	<0.001
Extrinsic Motivation ↔ Intrinsic Motivation	0.486	0.062	7.796	<0.001

## Data Availability

The data presented in this study are available on request from the corresponding author.

## Appendix A

**Table A1:** Measure Items.

Variable	Code	Description
Perceived Behavioral Control	PBC1	My financial capabilities do not hinder my desire to pursue a professional playing career.
	PBC2	I am confident in overcoming problems that may arise while pursuing a professional playing career.
	PBC3	I can balance other commitments while simultaneously striving to have a professional playing career.
	PBC4	My dedication and effort increase my chances of being a professional player in my sport.
	PBC5	I have the necessary skills needed to ensure the pursuance of my professional playing career.
Subjective Norm	SN1	I am greatly influenced by my coaches/mentors on my decision to pursue a professional playing career.
	SN2	I do not feel/receive pressure to pursue a professional playing career.
	SN3	Professional experts have a great impact on my decision to pursue a professional playing career.
	SN4	I am greatly influenced by my family on my decision to pursue a professional playing career.
	SN5	The public’s perception has a great impact on my decision to pursue a professional playing career.
	SN6	Seeing professional players inspired me to pursue a professional playing career.
Affective Behavior (Attitude)	AB1	I get excited whenever I envision myself as a professional player.
	AB2	I am very eager to pursue a professional playing career.
	AB3	I feel passionate about dedicating my life to be a professional player.
	AB4	I am excited to experience the challenges and growth opportunities of a professional playing career.
	AB5	I will be greatly satisfied when I become a professional player.
Extrinsic Motivation	EM1	Money and fame help me to be dedicated in pursuing a professional playing career.
	EM2	I feel motivated by the goals set by my coach in pursuing a professional playing career.
	EM3	Recognitions and rewards help me to stay motivated as a player.
	EM4	My desire to please my coaches and teammates helps me to keep motivated.
	EM5	A potential large fanbase or following in social media helps me to keep motivated.
Intrinsic Motivation	IM1	My dedication to pursue a professional playing career is greatly driven by my passion and love for my sport.
	IM2	I feel joyous and satisfied during training and while improving my skills.
	IM3	I am greatly motivated based solely on my love for my sport, without considering any other influence.
	IM4	My commitment and effort in my sport is greatly influenced by my satisfaction whenever I achieve personal goals.
	IM5	I am greatly passionate about my current sport.
Self-Efficacy	SE1	I trust myself to perform consistently in my sport.
	SE2	I am capable of performing and developing the necessary skills of a professional player.
	SE3	I am confident to handle pressure and demands of a professional playing career.
	SE4	I am confident in my ability to decide on critical decisions concerning my professional playing career.
	SE5	I believe in my ability to overcome certain setbacks and challenges in pursuing a professional playing career.
Attitude	A1	I have a positive mindset during training sessions.
	A2	I am optimistic that my hard work and dedication will pay off.
	A3	I am greatly motivated by my long-term goals to constantly improve my skills.
	A4	My sport greatly enhances my mental and physical well-being.
	A5	I focus on upholding a healthy and balanced lifestyle as a player.
Pursuance Intention	PI1	I am planning to pursue a professional playing career.
	PI2	I will try to continue professional playing as my career.
	PI3	I prepare and actively plan my steps in becoming a professional athlete.
	PI4	I am committed to actively seeking any opportunities for a professional career.
	PI5	I will be satisfied if I become a professional player.

## Appendix B

**Table A2:** ANOVA Results.

		Sum of Squares	df	Mean Square	F	Sig.
PBC1	Between Groups	60.308	2	30.154	35.298	0.000
	Within Groups	297.282	348	0.854		
	Total	357.590	350			
PBC2	Between Groups	75.124	2	37.562	50.204	0.000
	Within Groups	260.369	348	0.748		
	Total	335.493	350			
PBC3	Between Groups	109.356	2	54.678	66.230	0.000
	Within Groups	287.300	348	0.826		
	Total	396.655	350			
PBC4	Between Groups	63.642	2	31.821	36.874	0.000
	Within Groups	300.313	348	0.863		
	Total	363.954	350			
PBC5	Between Groups	133.808	2	66.904	74.358	0.000
	Within Groups	313.115	348	0.900		
	Total	446.923	350			
SN1	Between Groups	143.660	2	71.830	84.302	0.000
	Within Groups	296.516	348	0.852		
	Total	440.177	350			
SN2	Between Groups	113.920	2	56.960	56.514	0.000
	Within Groups	350.746	348	1.008		
	Total	464.667	350			
SN3	Between Groups	153.383	2	76.691	98.045	0.000
	Within Groups	272.207	348	0.782		
	Total	425.590	350			
SN4	Between Groups	176.155	2	88.078	86.085	0.000
	Within Groups	356.056	348	1.023		
	Total	532.211	350			
SN5	Between Groups	191.289	2	95.645	92.576	0.000
	Within Groups	359.537	348	1.033		
	Total	550.826	350			
SN6	Between Groups	152.722	2	76.361	90.276	0.000
	Within Groups	294.360	348	0.846		
	Total	447.083	350			
AF1	Between Groups	105.205	2	52.602	76.340	0.000
	Within Groups	239.792	348	0.689		
	Total	344.997	350			
AF2	Between Groups	214.885	2	107.443	163.927	0.000
	Within Groups	228.089	348	0.655		
	Total	442.974	350			
AF3	Between Groups	201.846	2	100.923	136.578	0.000
	Within Groups	257.151	348	0.739		
	Total	458.997	350			
AF4	Between Groups	162.517	2	81.259	116.145	0.000
	Within Groups	243.471	348	0.700		
	Total	405.989	350			
AF5	Between Groups	182.524	2	91.262	143.549	0.000
	Within Groups	221.243	348	0.636		
	Total	403.766	350			
EX1	Between Groups	109.371	2	54.686	50.385	0.000
	Within Groups	377.706	348	1.085		
	Total	487.077	350			
EX2	Between Groups	190.762	2	95.381	125.382	0.000
	Within Groups	264.731	348	0.761		
	Total	455.493	350			
EX3	Between Groups	134.231	2	67.115	75.800	0.000
	Within Groups	308.128	348	0.885		
	Total	442.359	350			
EX4	Between Groups	136.041	2	68.021	75.494	0.000
	Within Groups	313.549	348	0.901		
	Total	449.590	350			
EX5	Between Groups	190.377	2	95.188	78.975	0.000
	Within Groups	419.441	348	1.205		
	Total	609.818	350			
IM1	Between Groups	129.764	2	64.882	111.530	0.000
	Within Groups	202.447	348	0.582		
	Total	332.211	350			
IM2	Between Groups	103.126	2	51.563	73.246	0.000
	Within Groups	244.982	348	0.704		
	Total	348.108	350			
IM3	Between Groups	183.243	2	91.621	132.046	0.000
	Within Groups	241.464	348	0.694		
	Total	424.707	350			
IM4	Between Groups	115.669	2	57.834	85.645	0.000
	Within Groups	234.998	348	0.675		
	Total	350.667	350			
IM5	Between Groups	197.589	2	98.795	125.476	0.000
	Within Groups	274.000	348	0.787		
	Total	471.590	350			
SE1	Between Groups	131.574	2	65.787	102.503	0.000
	Within Groups	223.349	348	0.642		
	Total	354.923	350			
SE2	Between Groups	172.649	2	86.324	131.600	0.000
	Within Groups	228.274	348	0.656		
	Total	400.923	350			
SE3	Between Groups	165.519	2	82.759	127.222	0.000
	Within Groups	226.378	348	0.651		
	Total	391.897	350			
SE4	Between Groups	186.344	2	93.172	128.895	0.000
	Within Groups	251.553	348	0.723		
	Total	437.897	350			
SE5	Between Groups	192.218	2	96.109	125.025	0.000
	Within Groups	267.514	348	0.769		
	Total	459.732	350			
AT1	Between Groups	106.068	2	53.034	94.855	0.000
	Within Groups	194.570	348	0.559		
	Total	300.638	350			
AT2	Between Groups	89.681	2	44.841	71.661	0.000
	Within Groups	217.755	348	0.626		
	Total	307.436	350			
AT3	Between Groups	117.191	2	58.596	98.137	0.000
	Within Groups	207.783	348	0.597		
	Total	324.974	350			
AT4	Between Groups	90.950	2	45.475	58.362	0.000
	Within Groups	271.158	348	0.779		
	Total	362.108	350			
AT5	Between Groups	163.779	2	81.890	114.808	0.000
	Within Groups	248.221	348	0.713		
	Total	412.000	350			
PI1	Between Groups	219.468	2	109.734	152.238	0.000
	Within Groups	250.840	348	0.721		
	Total	470.308	350			
PI2	Between Groups	267.269	2	133.634	201.241	0.000
	Within Groups	231.090	348	0.664		
	Total	498.359	350			
PI3	Between Groups	273.163	2	136.582	196.778	0.000
	Within Groups	241.543	348	0.694		
	Total	514.707	350			
PI4	Between Groups	249.633	2	124.816	181.258	0.000
	Within Groups	239.638	348	0.689		
	Total	489.271	350			
PI5	Between Groups	259.481	2	129.741	192.269	0.000
	Within Groups	234.826	348	0.675		
	Total	494.308	350			

## Appendix C

**Table A3:** Descriptive Statistics of Three-Cluster Output.

		N	Mean	Std. Deviation	Std. Error	95% Confidence Interval for Mean	
						Lower Bound	Upper Bound
PBC1	1	186	4.3656	0.76101	0.05580	4.2555	4.4757
	2	102	3.9118	0.99606	0.09862	3.7161	4.1074
	3	63	3.2540	1.20440	0.15174	2.9506	3.5573
	Total	351	4.0342	1.01078	0.05395	3.9281	4.1403
PBC2	1	186	4.4247	0.73318	0.05376	4.3187	4.5308
	2	102	3.9608	0.86656	0.08580	3.7906	4.1310
	3	63	3.1746	1.17143	0.14759	2.8796	3.4696
	Total	351	4.0655	0.97906	0.05226	3.9627	4.1683
PBC3	1	186	4.4624	0.73608	0.05397	4.3559	4.5688
	2	102	3.9118	0.96578	0.09563	3.7221	4.1015
	3	63	2.9524	1.22380	0.15418	2.6442	3.2606
	Total	351	4.0313	1.06457	0.05682	3.9196	4.1431
PBC4	1	186	4.3226	0.79405	0.05822	4.2077	4.4374
	2	102	3.8824	0.91515	0.09061	3.7026	4.0621
	3	63	3.1746	1.26414	0.15927	2.8562	3.4930
	Total	351	3.9886	1.01974	0.05443	3.8816	4.0957
PBC5	1	186	4.5806	0.76154	0.05584	4.4705	4.6908
	2	102	3.8627	1.05342	0.10430	3.6558	4.0697
	3	63	2.9365	1.22965	0.15492	2.6268	3.2462
	Total	351	4.0769	1.13001	0.06032	3.9583	4.1955
SN1	1	186	4.4892	0.65956	0.04836	4.3938	4.5847
	2	102	3.6765	1.07318	0.10626	3.4657	3.8873
	3	63	2.8095	1.26819	0.15978	2.4901	3.1289
	Total	351	3.9516	1.12145	0.05986	3.8338	4.0693
SN2	1	186	4.4140	0.76095	0.05580	4.3039	4.5241
	2	102	3.4314	1.18162	0.11700	3.1993	3.6635
	3	63	3.0794	1.28643	0.16207	2.7554	3.4033
	Total	351	3.8889	1.15222	0.06150	3.7679	4.0098
SN3	1	186	4.5806	0.50559	0.03707	4.5075	4.6538
	2	102	3.3922	1.09143	0.10807	3.1778	3.6065
	3	63	3.0794	1.29890	0.16365	2.7522	3.4065
	Total	351	3.9658	1.10271	0.05886	3.8501	4.0816
SN4	1	186	4.4516	0.82549	0.06053	4.3322	4.5710
	2	102	3.3039	1.14138	0.11301	3.0797	3.5281
	3	63	2.7302	1.25988	0.15873	2.4129	3.0475
	Total	351	3.8091	1.23313	0.06582	3.6797	3.9386
SN5	1	186	4.4785	0.77951	0.05716	4.3657	4.5913
	2	102	3.2647	1.20991	0.11980	3.0271	3.5024
	3	63	2.6984	1.26536	0.15942	2.3797	3.0171
	Total	351	3.8063	1.25451	0.06696	3.6746	3.9380
SN6	1	186	4.6882	0.49818	0.03653	4.6161	4.7602
	2	102	3.6275	1.16824	0.11567	3.3980	3.8569
	3	63	3.0794	1.33564	0.16827	2.7430	3.4157
	Total	351	4.0912	1.13021	0.06033	3.9725	4.2098
AF1	1	186	4.5269	0.56168	0.04118	4.4456	4.6081
	2	102	4.1569	0.81745	0.08094	3.9963	4.3174
	3	63	3.0317	1.35561	0.17079	2.6903	3.3732
	Total	351	4.1510	0.99283	0.05299	4.0468	4.2552
AF2	1	186	4.5484	0.57921	0.04247	4.4646	4.6322
	2	102	3.9510	0.99879	0.09889	3.7548	4.1472
	3	63	2.4127	1.02603	0.12927	2.1543	2.6711
	Total	351	3.9915	1.12501	0.06005	3.8734	4.1096
AF3	1	186	4.5699	0.60445	0.04432	4.4825	4.6573
	2	102	3.8922	0.99411	0.09843	3.6969	4.0874
	3	63	2.5079	1.20313	0.15158	2.2049	2.8109
	Total	351	4.0028	1.14517	0.06112	3.8826	4.1231
AF4	1	186	4.5161	0.60828	0.04460	4.4281	4.6041
	2	102	3.9020	0.99015	0.09804	3.7075	4.0964
	3	63	2.6667	1.10716	0.13949	2.3878	2.9455
	Total	351	4.0057	1.07702	0.05749	3.8926	4.1188
AF5	1	186	4.7312	0.50167	0.03678	4.6586	4.8038
	2	102	4.2157	0.90791	0.08990	4.0374	4.3940
	3	63	2.7619	1.21435	0.15299	2.4561	3.0677
	Total	351	4.2279	1.07407	0.05733	4.1152	4.3407
EX1	1	186	4.0484	1.03601	0.07596	3.8985	4.1983
	2	102	3.8431	0.98247	0.09728	3.6502	4.0361
	3	63	2.5397	1.14758	0.14458	2.2507	2.8287
	Total	351	3.7179	1.17968	0.06297	3.5941	3.8418
EX2	1	186	4.4570	0.77862	0.05709	4.3444	4.5696
	2	102	3.9020	0.95968	0.09502	3.7135	4.0905
	3	63	2.4444	0.98009	0.12348	2.1976	2.6913
	Total	351	3.9345	1.14079	0.06089	3.8147	4.0542
EX3	1	186	4.3495	0.85830	0.06293	4.2253	4.4736
	2	102	4.0392	0.88912	0.08804	3.8646	4.2139
	3	63	2.6667	1.21814	0.15347	2.3599	2.9735
	Total	351	3.9573	1.12423	0.06001	3.8392	4.0753
EX4	1	186	4.3817	0.86336	0.06330	4.2568	4.5066
	2	102	4.0000	0.92276	0.09137	3.8188	4.1812
	3	63	2.6825	1.20249	0.15150	2.3797	2.9854
	Total	351	3.9658	1.13338	0.06050	3.8468	4.0848
EX5	1	186	4.3763	1.06455	0.07806	4.2223	4.5303
	2	102	3.5882	1.14626	0.11350	3.3631	3.8134
	3	63	2.3968	1.11500	0.14048	2.1160	2.6776
	Total	351	3.7920	1.31998	0.07046	3.6535	3.9306
IM1	1	186	4.6129	0.55082	0.04039	4.5332	4.6926
	2	102	4.1863	0.74102	0.07337	4.0407	4.3318
	3	63	2.9524	1.21055	0.15252	2.6475	3.2573
	Total	351	4.1909	0.97426	0.05200	4.0886	4.2932
IM2	1	186	4.5914	0.57397	0.04209	4.5084	4.6744
	2	102	4.2255	0.79487	0.07870	4.0694	4.3816
	3	63	3.1111	1.39250	0.17544	2.7604	3.4618
	Total	351	4.2194	0.99729	0.05323	4.1147	4.3241
IM3	1	186	4.6237	0.52841	0.03875	4.5472	4.7001
	2	102	4.1569	0.82947	0.08213	3.9939	4.3198
	3	63	2.6508	1.39306	0.17551	2.3000	3.0016
	Total	351	4.1339	1.10157	0.05880	4.0183	4.2495
IM4	1	186	4.5323	0.58958	0.04323	4.4470	4.6175
	2	102	4.0490	0.86032	0.08518	3.8800	4.2180
	3	63	2.9683	1.24393	0.15672	2.6550	3.2815
	Total	351	4.1111	1.00095	0.05343	4.0060	4.2162
IM5	1	186	4.7527	0.59103	0.04334	4.6672	4.8382
	2	102	4.0686	0.96739	0.09579	3.8786	4.2586
	3	63	2.7143	1.36108	0.17148	2.3715	3.0571
	Total	351	4.1880	1.16078	0.06196	4.0662	4.3099
SE1	1	186	4.5645	0.51845	0.03801	4.4895	4.6395
	2	102	3.9020	0.86184	0.08533	3.7327	4.0712
	3	63	2.9206	1.26110	0.15888	2.6030	3.2382
	Total	351	4.0769	1.00701	0.05375	3.9712	4.1826
SE2	1	186	4.6237	0.50754	0.03721	4.5502	4.6971
	2	102	3.9118	0.93452	0.09253	3.7282	4.0953
	3	63	2.7302	1.22087	0.15382	2.4227	3.0376
	Total	351	4.0769	1.07028	0.05713	3.9646	4.1893
SE3	1	186	4.5215	0.56192	0.04120	4.4402	4.6028
	2	102	3.9412	0.88802	0.08793	3.7668	4.1156
	3	63	2.6508	1.19351	0.15037	2.3502	2.9514
	Total	351	4.0171	1.05816	0.05648	3.9060	4.1282
SE4	1	186	4.6022	0.55292	0.04054	4.5222	4.6821
	2	102	3.7941	0.96819	0.09587	3.6039	3.9843
	3	63	2.6508	1.27202	0.16026	2.3304	2.9711
	Total	351	4.0171	1.11854	0.05970	3.8997	4.1345
SE5	1	186	4.7473	0.54586	0.04002	4.6683	4.8263
	2	102	3.7941	1.07481	0.10642	3.5830	4.0052
	3	63	2.8095	1.24249	0.15654	2.4966	3.1224
	Total	351	4.1225	1.14609	0.06117	4.0022	4.2428
AT1	1	186	4.5806	0.55649	0.04080	4.5001	4.6611
	2	102	4.2059	0.63462	0.06284	4.0812	4.3305
	3	63	3.0794	1.24825	0.15726	2.7650	3.3937
	Total	351	4.2023	0.92680	0.04947	4.1050	4.2996
AT2	1	186	4.5860	0.55568	0.04074	4.5056	4.6664
	2	102	4.2745	0.69163	0.06848	4.1387	4.4104
	3	63	3.2063	1.34595	0.16957	2.8674	3.5453
	Total	351	4.2479	0.93722	0.05003	4.1495	4.3463
AT3	1	186	4.5914	0.55481	0.04068	4.5111	4.6717
	2	102	4.2647	0.65893	0.06524	4.1353	4.3941
	3	63	3.0159	1.31360	0.16550	2.6850	3.3467
	Total	351	4.2137	0.96359	0.05143	4.1125	4.3148
AT4	1	186	4.5645	0.56819	0.04166	4.4823	4.6467
	2	102	4.2353	0.92465	0.09155	4.0537	4.4169
	3	63	3.1746	1.42036	0.17895	2.8169	3.5323
	Total	351	4.2194	1.01715	0.05429	4.1126	4.3262
AT5	1	186	4.8172	0.45193	0.03314	4.7518	4.8826
	2	102	4.3039	0.84184	0.08335	4.1386	4.4693
	3	63	2.9524	1.49654	0.18855	2.5755	3.3293
	Total	351	4.3333	1.08496	0.05791	4.2194	4.4472
PI1	1	186	4.4731	0.58985	0.04325	4.3878	4.5584
	2	102	3.8235	0.99913	0.09893	3.6273	4.0198
	3	63	2.3175	1.17536	0.14808	2.0215	2.6135
	Total	351	3.8974	1.15920	0.06187	3.7757	4.0191
PI2	1	186	4.5914	0.56447	0.04139	4.5097	4.6731
	2	102	3.7843	0.98128	0.09716	3.5916	3.9771
	3	63	2.2222	1.09904	0.13847	1.9454	2.4990
	Total	351	3.9316	1.19327	0.06369	3.8064	4.0569
PI3	1	186	4.5699	0.52808	0.03872	4.4935	4.6463
	2	102	3.7549	1.08488	0.10742	3.5418	3.9680
	3	63	2.1746	1.07072	0.13490	1.9049	2.4443
	Total	351	3.9031	1.21268	0.06473	3.7758	4.0304
PI4	1	186	4.5806	0.52654	0.03861	4.5045	4.6568
	2	102	3.8431	0.99250	0.09827	3.6482	4.0381
	3	63	2.2857	1.19715	0.15083	1.9842	2.5872
	Total	351	3.9544	1.18234	0.06311	3.8303	4.0785
PI5	1	186	4.8763	0.34607	0.02537	4.8263	4.9264
	2	102	4.0980	1.05783	0.10474	3.8903	4.3058
	3	63	2.5397	1.26778	0.15973	2.2204	2.8590
	Total	351	4.2308	1.18841	0.06343	4.1060	4.3555
